# Web-Based Survival Analysis Tool Tailored for Medical Research (KMplot): Development and Implementation

**DOI:** 10.2196/27633

**Published:** 2021-07-26

**Authors:** András Lánczky, Balázs Győrffy

**Affiliations:** 1 Department of Bioinformatics Semmelweis University Budapest Hungary; 2 TTK Lendület Cancer Biomarker Research Group Institute of Enzymology Research Centre for Natural Sciences Budapest Hungary

**Keywords:** Kaplan-Meier plot, internet, Cox regression, follow-up, multivariate analysis, survival

## Abstract

**Background:**

Survival analysis is a cornerstone of medical research, enabling the assessment of clinical outcomes for disease progression and treatment efficiency. Despite its central importance, no commonly used spreadsheet software can handle survival analysis and there is no web server available for its computation.

**Objective:**

Here, we introduce a web-based tool capable of performing univariate and multivariate Cox proportional hazards survival analysis using data generated by genomic, transcriptomic, proteomic, or metabolomic studies.

**Methods:**

We implemented different methods to establish cut-off values for the trichotomization or dichotomization of continuous data. The false discovery rate is computed to correct for multiple hypothesis testing. A multivariate analysis option enables comparing omics data with clinical variables.

**Results:**

We established a registration-free web-based survival analysis tool capable of performing univariate and multivariate survival analysis using any custom-generated data.

**Conclusions:**

This tool fills a gap and will be an invaluable contribution to basic medical and clinical research.

## Introduction

Bioinformatic programs include databases, algorithms, services, and software tools. These not only span a wide range of utility but have also gained increased value in scientific research in recent years; approximately 80% of papers published in biology and 60% of papers published in medicine report the use of at least one bioinformatic tool [1]. We recently analyzed the landscape of web-based bioinformatic services and uncovered 3649 such publications since 1994, 69% of which are actively maintained [2]. The leading advantages of browser-based bioinformatic programs include unrestricted availability, the lack of need for the installation of specific software packages, optimized allocation of computational resources, the possibility of constant updates, instant access to the latest versions, and the opportunity to enable real-time validation of previous analysis results. A subcohort of these tools enables certain analyses with user-provided data. A few representative examples used in these tools in medical research include an online calculator for receiver operator characteristics [3], a tool to determine optimal cut-off values for clinical tests [4], and a sample size calculator for randomized clinical trials [5].

The assessment of survival following the onset of a disease or of a treatment is a fundamental analysis in medical research. In an optimal scenario, the differential survival of two cohorts can be compared by employing a simple Mann-Whitney test. However, survival times do not follow a normal distribution and it is common for numerous subjects to lack associated event data at the end of follow-up. Kaplan and Meier [6] proposed a simple and elegant solution to these issues by including all cases regardless of endpoint status in the analysis. The basic concept of Kaplan-Meier survival analysis is to assign a “censored” status to incomplete observations at the end of the follow-up time. In other words, there are two inputs for each case: the length of the follow-up time and a binary classifier designating the case as one with an event or one that is censored. Then, starting from 100, at each event, the survival line drops in proportion to the number of samples remaining within the investigated cohort. If there are multiple survival curves, the statistical difference between these is most commonly computed by employing the Cox proportional-hazards regression model [7].

Despite its widespread use, there is no online tool available for survival analysis. Therefore, it is necessary to acquire specialized software packages, as none of the general office packages (OpenOffice, LibreOffice, MS Office) is suitable for analyzing follow-up data. We previously established an online platform capable of linking survival outcome in various cancer types to mRNA [8] and microRNA [9] expression alterations. Here, we aimed to establish a freely available, easy-to-use online platform capable of performing survival analysis and constructing a Kaplan-Meier plot with any type of user-uploaded custom data containing any type of genomic or clinical information.

## Methods

### Setup of the Web Platform

The website is built on an Apache 2.4 web server and hosted by a Linux-based server machine. The user interface is written in PHP 7 and JavaScript using JQuery. The backend side is written in PHP 7 and R, and the repository layer is built on the PostgreSQL 12 database. The database temporarily contains the uploaded data and generated results. The analysis platform is accessible via any standard browser (Firefox, Edge, Chrome, Safari).

### Survival Analysis

Multiple R packages are used for the statistical computations and for generating the output graphs. The *survival* package [10] is used for univariate Kaplan-Meier analysis and the multivariate analysis. The survival curve and the beeswarm plot are generated by the *survplot* [11] and *beeswarm* [12] packages, respectively. The *XML* and *rjson* R packages are used to load the configuration files, the *RODBC* package is used to communicate with the database, and the *ggplot2* package [13] is used to visualize the results.

**Figure 1:**
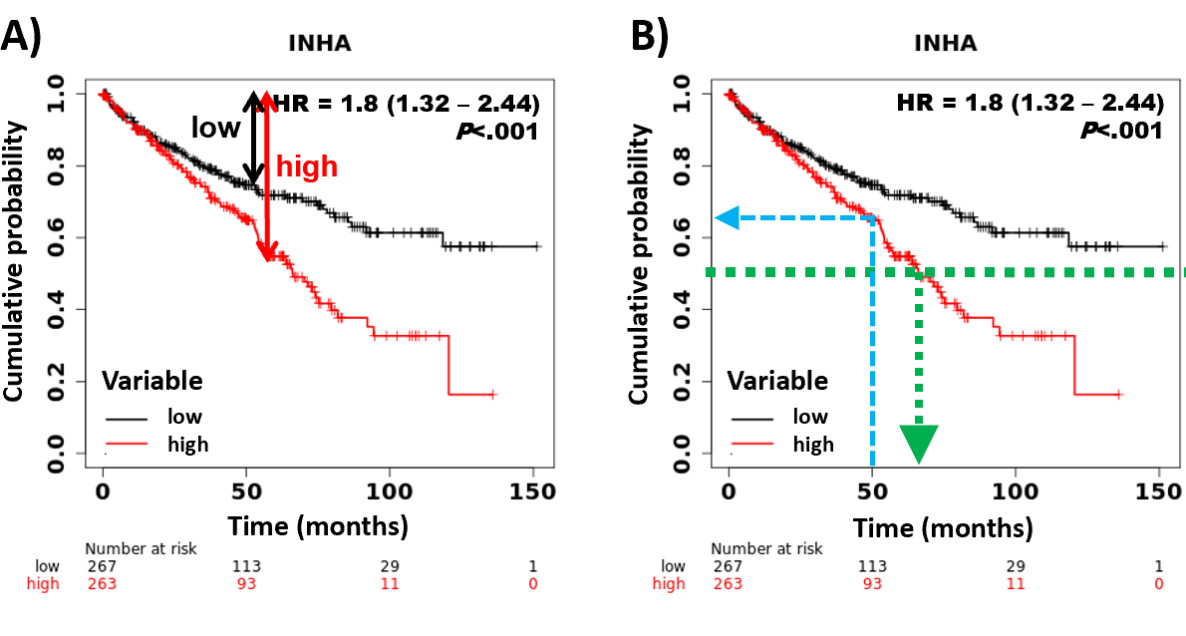
Kaplan-Meier curves showing main concepts used in survival analysis, including the (A) hazard rate (high/low) and (B) median survival. The green arrow shows the visually determined median survival and the blue arrow shows the survival probability at 50 months.

When comparing two cohorts, the significance is computed using the Cox-Mantel (log rank) test [7]. The difference between the cohorts is numerically characterized by the hazard rate (HR), which is based on the differential descent rate of the two cohorts (Figure 1A). Of note, since the hazard rate is by definition a comparison to the baseline, a relative two-fold drop in one cohort is equal to a half-fold drop in the other cohorts. Basically, depending on the context, an HR of 2 equals an HR of 0.5. As it is easier to understand an HR value above 1 in most cases, we implemented an option to invert all HR values below 1.

The generated results also include the median survival time, which is the time at which the probability of 0.5 is reached in one of the cohorts. The median time can also be determined visually by drawing a vertical line from the selected probability to the X axis. Of note, performing the steps backward can determine the cumulative probability of survival at a given time point (Figure 1B).

### Assigning the Samples into Two Cohorts

To enable visualization in the Kaplan-Meier plot, it is necessary to establish a cut-off value and assign the samples to one of two cohorts. We implemented three different options for this task: using a predefined quantile (including the median, upper, and lower quartiles), trichotomizing the data (eg, assign the data into three cohorts and then omit the middle cohort), and using the best available cut-off value.

**Figure 2:**
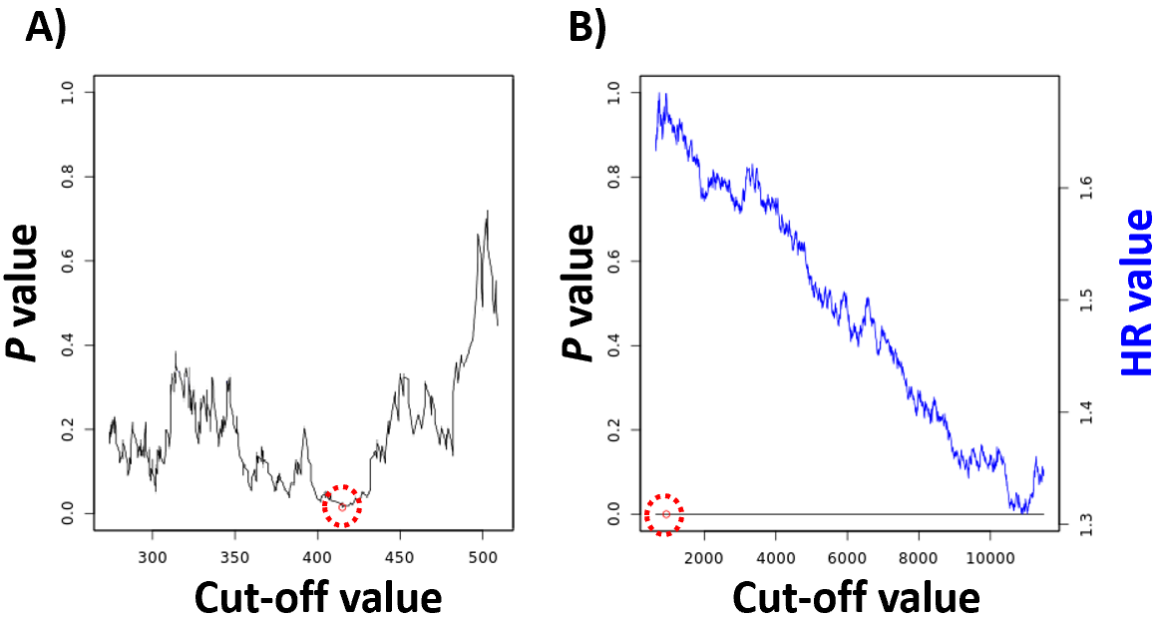
A cut-off plot can be used to visualize the correlation between the used cut-off values and the achieved *P* values (A) and hazard rate (HR) (B). The red circle identifies the best cutoff. The computation of false discovery rate across all *P* values provides correction for multiple hypothesis testing.

To find the best cutoff, we iterate over the input variable values from the lower quartile to the upper quartile and compute the Cox regression [7] for each setting. The most significant cut-off value is used as the best cutoff to separate the input data into two groups. We implemented a simple visual representation of this analysis by showing the achieved *P* values in relation to the used cut-off values (Figure 2A). In case the generated cut-off values are ambiguous (eg, multiple cut-off values deliver very low *P* values), the cut-off value corresponding to the highest HR is used (see Figure 2B).

### Quality Control

During the computation of multiple cut-off values, multiple hypotheses are generated. Therefore, the false discovery rate (FDR) is computed by default in this setting using the Benjamini-Hochberg method [14] to correct for multiple hypothesis testing. The FDR results are normally shown in the “Results” page.

A requirement for Cox regression is that the hazard is independent of time. To fulfill this requirement, the censoring should be independent of the prognosis, samples entering at different time points in the analysis should have the same prognosis, and the time should be measured as a continuous variable (not in bins). We employed the coxph function of the *survival* package [10] for performing the proportional hazard assumption test.

In some cases, one might want to compare clinical and genomic variables. To enable this, clinical data can be selected not only as filters but also as variables to be included in the multivariate analysis. In these analyses, the “Results” page displays the *P* values and HR values for each variable included in the multivariate analysis in a table format.

### Using Multiple Variables

We implemented multiple options to simultaneously use and combine multiple variables. Each of these settings uses the original variable values as input and basic mathematical functions to calculate the new joint values.

The simplest option is to select multiple variables and then use each variable separately. In this case, the same analysis is performed for each selected marker using the exact same filtering settings. This option is identical to running the analysis for each variable consecutively.

In the second feature, one can use the mean expression of a panel of variables; in this case, any variable can be inverted and a weight can be added to each. Using the mean expression of a set of genes can be termed a “signature analysis,” as the expression of each included variable will influence the value of the final “composite variable.” This feature can also be used to validate previously published gene expression signatures utilizing a preselected panel of genes.

A third option is utilization of the ratio of two genes; in this case, one variable is used as the numerator, the other variable is used as the denominator, and a new value is computed for each sample. This setting is useful when one wants to compare the expression values to a reference gene such as *GAPDH*.

The fourth option enables the stratification of all patients based on the median expression level of a selected variable and then use another variable in the high or low cohort only. This enables the investigation of a selected variable in an already stratified cohort and ultimately the setup of a decision tree–like classification for the investigated cohort.

In each of the settings where multiple variables are combined, a new value based on the equation is generated for each sample, which is then used when performing the survival analysis, including the cut-off selection. Of note, one might want to directly compare two or more selected continuous variables to each other. For this purpose, we implemented an option to compute Spearman and Pearson correlation coefficients between the variables using the *cor.test* function from the basic R distribution.

## Results

We established an online survival analysis platform that grabs a user-generated tab-separated or semicolon-separated file as input. The table headers can include case-insensitive letters of the English alphabet, numbers, spaces, underscores, colons, round brackets, and exclamation marks as characters. The content within the table cells can be numeric or text values. Some columns can be used as filters and a maximum of three filters are allowed. Table 1 provides a quick guide for the setup of an input file. The file can be a comma-separated value or a tab-separated table, and different types of data are allowed in each column. Table 2 shows a sample input file using this guide; the maximal dimensions of the table are 100 columns and 8000 rows. Note that a gene can be in the form of text when used as a group. Using this data table, the system is capable of performing univariate and multivariate survival analysis by using one or multiple variables and clinical data. In addition to drawing a Kaplan-Meier plot, the *P* values and HR values with 95% CIs are also computed. A separate plot visualizes the correlation between the *P* values and HR values and the employed cut-off values. Median survival values are computed for cohorts reaching a cumulative probability of 0.5, and upper-quartile survival is computed for the remaining cases. Of note, when performing multivariate analysis, only patient samples for which all variables of interest are concurrently available can be included. The platform includes multiple quality-control steps, including validation of the proportional hazard assumption and computation of the FDR for cases where multiple analyses are run simultaneously. The web service is freely available without requiring registration [15].

**Table 1:** Quick start guide for setting up an input file.

Header name	Sample ID	Survival time	Survival event	Filter	Gene
Automatically recognized	Yes	Yes	Yes	Yes	No
Maximal number of different values	No limit	No limit	2 (0 or 1)	10	No limit
Can be text	Yes	No	No	Yes	No
Can be binary	No	No	Yes	Yes	Yes
Can be continuous		Yes	No	No	Yes

**Table 2:** Sample input file.

Sample ID	Survival time	Survival event	Filter_A	Filter_B	Filter_C	Gene_1	ABC123	DE45
Sample 1	95	1	2	2	3	1441	4474	1.13
Sample 2	66	0	3	3	3	3064	421	2.395
Sample 3	70	0	3	1	1	2529	2974	1.363
Sample 4	26	1	3	1	3	19	3346	4.818
Sample 5	13	0	1	2	3	3573	1244	2.058
Sample 6	67	0	2	3	2	2977	962	4.431
Sample 7	96	1	3	3	3	2777	4367	2.015
Sample 8	67	0	3	3	1	4606	4190	1.05
Sample 9	95	1	3	1	2	1209	3930	1.980
Sample 10	1	1	2	3	2	1894	4897	4.073

## Discussion

Currently, genomics, transcriptomics, proteomics, and metabolomics enable the simultaneous investigation of multiple markers related to patient prognosis in experimental and clinical studies. Multiple online tools make survival analysis possible using previously published datasets such as those employing data from The Cancer Genome Atlas [9,16]. Despite the almost ubiquitous use of Cox regression to correlate different marker levels to prognosis, there is no available software to perform survival analysis for user-generated custom datasets. We established a wide-ranging online tool capable of performing Cox regression and constructing Kaplan-Meier plots for user-generated data. A comprehensive and practical review of the Kaplan-Meier curves has been published previously [17].

A major advantage of our platform is the inclusion of multiple choices to select a cut-off value to be used in the analysis. To generate a Kaplan-Meier plot, one must first determine a cutoff; a convenient and widespread option for this task is the median expression value [18,19]. However, the cutoff should be based on the intention of the study. In most medical studies, there is no biological reason that a certain predetermined quantile cutoff should discriminate two cohorts [20]. When a researcher aims to uncover any potential correlation between a variable and outcome, then all possible cut-off values can be checked. Of course, in such cases, the chance of false-positive results also increases; therefore, we have implemented the Benjamini-Hochberg method [14] to calculate the FDR to correct for multiple hypothesis testing. Our approach is rather conservative as the different analyses are not truly independent in such a scenario, as only a few samples can switch cohorts between successive analyses. Of note, independent of the used cutoff, a single-variable analysis is almost never sufficient to prove a direct correlation and thus multivariate analysis should not be omitted.

The analysis automatically checks the proportional hazards assumption to evaluate the independence from time. This can also be achieved by a simple visual inspection of the graph: in case there seems to be a significant difference between the two cohorts but the lines cross at multiple time points, then the hazard is clearly not independent of time [21]. Of note, a common question is whether or not crossing at the right end of the plot violates the proportional hazards assumption. In most cases, at the end of the follow-up time, only few patients remain in both cohorts. Thus, because the drop in the line for each event is proportional to all samples remaining in the analysis, even an event for a single patient can result in crossing of the two lines. However, this will not affect the significance of the entire analysis.

When interpreting the results, one has to be aware of some common caveats of survival analysis. First, the *P* value should be interpreted with respect to the sample size. The Cox model is not suitable for small sample sizes (N<40), and in these cases the generalized log-rank method is a better choice [22]. Higher sample numbers will lead to better significance, even in cases where the HR values are lower. A representative example of this bias is the ill-fated FLEX phase III trial [23]. By investigating the effect of cetuximab in patients with advanced nonsmall cell lung cancer, the authors observed a difference in survival of 10.1 months vs 11.3 months in the untreated and treated cohort, respectively. Although this difference was initially considered to be sufficient to gain approval by the US Food and Drug Administration, the European Medicines Agency rejected approval of the drug. Their main problem with the trial was the minimal overall survival benefit of only 12% and that only the exceptionally high sample number (N=1125) helped to reach a minimally significant *P* value of .04 [23].

A second important deception is the proportion of recorded events within a study. As only the actual events contribute to the drops in survival curves, it is not possible to perform a meaningful survival analysis when the number of events is very low. This not only prevents the computation of median (or upper quartile) survival, but the accidental concentration of all events into one of the cohorts can lead to an infinite HR. For example, The Cancer Genome Atlas Network published a breast cancer dataset with approximately 1000 patient samples [24]. The authors had to note that because of the very short follow-up, only 11% of the samples had survival events, which prevented utilization of the dataset for survival analyses [24].

We also have to discuss some limitations of the software. The input file has to be carefully formatted, and a maximum of 100 columns and 8000 rows are allowed. Only full columns are acceptable as variables, a maximum of three filters can be defined, and the survival event can only be coded “0” or “1.” Although these restrictions can make the setup of the analysis challenging, when a correctly formatted table is uploaded, the system can automatically recognize columns representing a survival event or survival time. A second limitation is the exclusive use of the Cox proportional-hazards model to compute significance, and other tests such as the Cochran-Mantel-Haenszel test [25,26] or the Gehan-Breslow-Wilcoxon test [27,28] are not implemented. The reason for our restriction is the almost exclusive use of the Cox test in the current medical literature.

In summary, we established an online survival analysis tool capable of performing univariate and multivariate survival analysis using any custom-generated data. We believe that this registration-free online platform simultaneously integrating multiple different analysis and quality-control options will be a valuable tool for biomedical researchers.

## Abbreviations

FDR: false discovery rate
HR: hazard rate
